# Evaluation of cases of concussion and subluxation in the permanent dentition: a retrospective study

**DOI:** 10.1590/1678-7757-2017-0287

**Published:** 2018-04-18

**Authors:** Denise Pedrini, Sônia Regina Panzarini, Adelisa Rodolfo Ferreira Tiveron, Valéria Marisel de Abreu, Celso Koogi Sonoda, Wilson Roberto Poi, Daniela Atili Brandini

**Affiliations:** 1 Univ Estadual Paulista - UNESP, Faculdade de Odontologia de Araçatuba, Departmento de Cirurgia e Clínica Integrada, Araçatuba, São Paulo, Brasil.; 2 Univ Estadual Paulista - UNESP, Faculdade de Odontologia de Araçatuba, Programa de Pós-Graduação em Ciência Odontológica, Araçatuba, São Paulo, Brasil.

**Keywords:** Tooth injuries, Periodontal ligament, Wound healing

## Abstract

**Objectives:**

This study evaluated the evolution of cases of concussion and subluxation through a retrospective study of 20 years.

**Material and Methods:**

Were examined clinical and radiographic records of 1,309 patients who underwent treatment of dentoalveolar trauma in the discipline of Integrated Clinic of the School of Dentistry of Araçatuba, UNESP, of which we selected 137 whose patients had concussion and subluxation injuries, with average age of 23.3 (SD – 10.96). The variables collected were: gender, age, history of previous and actual trauma, treatments performed, the presence of necrotic pulp, and time elapsed until the same trauma. The concussion and subluxation groups were subjected to statistical analyses using the SPSS 16.0 version software (α=0.05), Chi-square, and t-tests.

**Results:**

Of the 301 teeth involved, 49 (16.3%) suffered concussion and 252 (83.7%), subluxation, being the upper anterior teeth the most affected (75.1%) for both conditions. Subluxation and concussion traumas were more prevalent in men aged 10 to 20 years, most caused by cycling accidents (36.2%). There was a concomitant presence of crown fracture in 21% of cases of concussion and 34.7% of subluxation. Pulp necrosis was detected in 16.3% (concussion) and 27.1% (subluxation) (p=0.12), and most occurred within 6 months after the trauma (p=0.29). The pulp necrosis shows a positive correlation with motorcycle accidents (p=0.01), direct impact (p≤0.0001), crown fracture with pulp exposure (p≤0.0001), darkening of the crown (p=0.004) and spontaneous pain (p≤0.0001); and negative correlation with indirect impact (p≤0.0001).

**Conclusions:**

Although concussion and subluxation traumas are considered of minor degrees, they must be monitored, since the possibility of pulp necrosis exists, and its early treatment favors a good prognosis.

## Introduction

Of the traumatic dental injuries of the periodontal tissues, concussion and subluxation present the less complex clinical resolution, not causing great emotional impact on patients and their families when occurring alone. Although the prognosis is favorable with little pulp and periodontal complications, it must always be monitored, since the possibility of pulp necrosis exists^9^.

Concussion is characterized by an injury of the tooth support structures without increased tooth mobility or tooth displacement, but with reaction to the horizontal or vertical percussion, and may be associated with crown fracture^3^ ^,^ ^15^. The pulp sensitivity test is usually positive and does not notice changes radiographically^7^ ^,^ ^9^ ^,^ ^16^. Subluxation already has increased mobility in the horizontal direction, and the tooth appears to be sensitive to percussion and occlusal forces, occurring or not bleeding from the gingival sulcus. Initially, sensitivity tests may be negative, but subsequently, they tend to respond positively and radiographically. Abnormalities are not found^3^ ^,^ ^7^ ^,^ ^15^ ^,^ ^16^, although a slight thickening of the periodontal ligament may be detected in cases of sharp mobility^9^. General characteristics of concussion and subluxation include edema and bleeding and breaking of some fibers of the periodontal ligament; moreover, the neurovascular pulp supply may be affected leading to necrotic pulp^3^. These injuries can go unnoticed by parents, and seeking care can occur only after the sequel have been installed, mainly when associated to severe traumas.

The literature has shown that dental trauma has increased and become a public health problem^1^. Retrospective studies can contribute to the knowledge of its repair process and possible complications, in addition to improve the prognosis. Thus, this study aimed to evaluate the evolution of cases of concussion and subluxation through a retrospective study of 20 years.

## Material and methods

The research protocol for this study was reviewed and approved by the local Research Ethics Committee (CAAE – 45716615.6.0000.5420). All participants received information about the objectives of the study and provided written informed consent regarding their participation. Were examined clinical and radiographic records of 1,309 patients who underwent dentoalveolar trauma treated by the group of the discipline of Integrated Clinic, School of Dentistry of Araçatuba, UNESP - Univ Estadual Paulista, over the period of 1992-2011. The study was conducted with a sample of 137 records from patients diagnosed with concussion injury and/or subluxation, average age of 23.3 (SD – 10.96).

**Figure 1:**
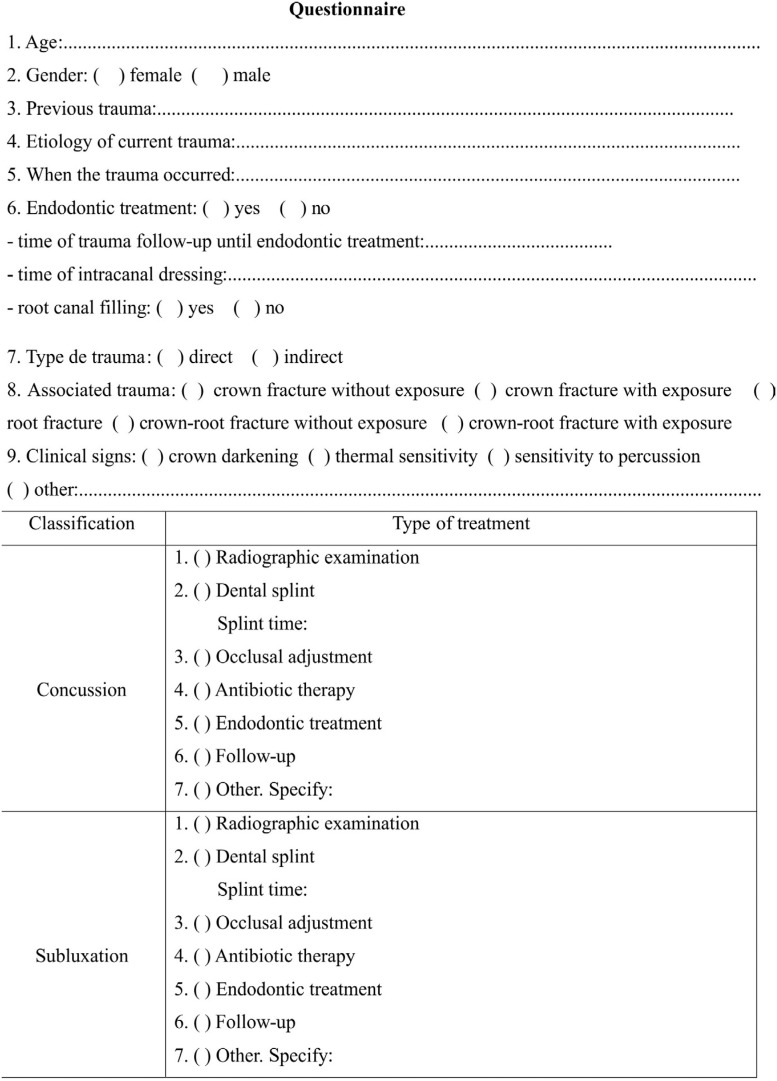
Questionnaire

All data were assessed by one trained dentist. The file assessment consisted of the following components: a) patient identification (gender and age); b) history of previous trauma; c) history of the actual trauma (etiology, type of trauma, clinical signs, and symptoms associated with dental trauma); d) treatments performed and; e) the presence of necrotic pulp and time elapsed until the same trauma (Figure 1).

The diagnosis of pulp necrosis was obtained through clinical and radiographic analysis. Clinical analysis was performed in all cases of both traumas, in addition to the monitoring of all patients using percussion testing and pulp sensitivity for a period of at least 24 months. Radiographic examination evaluated the increase of thickness of the periodontal ligament, radiolucent area compatible with periapical lesion, and presence of external root resorption.

Only permanent teeth were included in this sample. Were excluded the teeth that presented other causes than trauma, as caries and deep restoration, that could interfere in pulp status, pre-existing periodontal disease, and cases that had missing data.

After collection of data, the teeth were divided into concussion and subluxation groups and subjected to statistical analyses using the SPSS 16.0 version software (SPSS, Chicago, IL, USA) (α = 0.05). Group differences were analyzed using the Chi-square test for quantitative variables, Fisher's exact test for categorical variables, and independent *t*-tests for continuous variables. Nonparametric correlation (Spearman's rank correlation coefficient) was used to refer to a linear relation between two variables.

## Results

Concussion and subluxation traumas were more prevalent in men; concussion (49%) was the most prevalent type of injury among women, while subluxation (65.5%) (p=0.05) was the most prevalent among men aged 10 to 20 years (Table 1), most caused by cycling accidents (36.2%) (Table 3).

**Table 1:** Demographic characteristics and type of teeth in the cases of concussion and subluxation

Characteristics	Concussion		Subluxation		Total		P value
	n	%	n	%	n	%	
**Number of teeth**	49	16.3	252	83.7	301	100	
**Gender**							
Male	25	51	165	65.5	190	63.1	
Female	24	49	87	34.5	111	36.9	0.05*
**Age Group (Years)**							
Below 10	1	2.0	4	1.6	5	1.7	
10 to 20	31	63.3	140	55.6	171	56.8	
21 to 30	12	25	38	15.1	50	16.6	
31 to 40	3	6.1	46	18.3	49	16.3	
41 to 50	1	2.0	19	7.5	20	6.6	
Over 50	1	2.0	5	2.0	6	2.0	0.06
**Type of teeth**							
Upper Anterior	45	91.8	181	71.8	226	75.1	
Upper Posterior	0	0	3	1.2	3	1	
Lower Anterior	4	8.2	64	25.4	68	22.6	
Lower Posterior	4	0	4	1.6	4	1.3	0.003*

* denotes statistically significant result

P values are for comparisons between the two groups, and χ2 or Fisher's exact test for appropriate categorical variables

The occurrence of concussion and subluxation affected the anterior teeth more, but among the upper anterior teeth, concussion (91.8%) was the most prevalent type of injury, while subluxation (25.4%) (p=0.003) was the most prevalent among the lower teeth (Table 1). Presence of previous dental trauma occurred only in cases of subluxation (p=0.04) (Table 2).

**Table 2:** Dental history regarding previous and current dental traumas

History	Concussion		Subluxation		Total		P value
	n	%	n	%	n	%	
**Presence of previous dental trauma**							
No	48	100	229	92.3	277	93.6	
Yes	0	0	19	7.7	19	6.4	0.04*
**Associated dental trauma**							
No	32	65.3	198	78.6	230	76.4	
Crown fracture without pulp exposure	10	20.4	40	15.9	50	16.6	
Crown fracture with pulp exposure	7	14.3	13	5.2	20	6.6	
Root fracture	0	0	1	0.4	1	0.3	0.06

* denotes statistically significant result

P values are for comparisons between the two groups, and χ2 or Fisher's exact test for appropriate categorical variables

Motorcycle accidents represented the etiology of 20.4% of cases of concussions, and 7.9% (p=0.01) of cases of subluxation (Table 3), showing a positive association with concussion (correlation coefficient rank=0.128, p=0.027) and negative association with subluxation (correlation coefficient rank=-0.0124, p=0.032).

**Table 3:** Etiology and type of impact occurred regarding concussions and subluxations

Characteristics	Concussion		Subluxation		Total		P value
	n	%	n	%	n	%	
**Etiology**							
Fall from height	8	16.3	35	13.9	43	14.3	0.65
Ac. cycling	15	30.6	94	37.3	109	36.2	0.37
Ac. motorcycle	10	20.4	20	7.9	30	10	0.01*
Ac. automotive	2	4.1	34	13.5	36	12	0.06
Aggression	0	0	10	4	10	3.3	0.15
Sport	9	18.4	23	9.1	31	10.6	0.05*
Other	5	10.2	37	14.7	42	14	0.40
**Type of impact**							
Indirect	24	0	136	54	160	53.2	
Direct	25	51	112	44	137	45.5	0.50

* denotes statistically significant result

P values are for comparisons between the two groups, and χ2 or Fisher's exact test for appropriate categorical variables

Among the cases studied, 18.4% of concussion and 9.1% of subluxation cases happened during sports activities (p=0.05) (Table 3). Sporting accidents were also directly and significantly related to concussion (correlation coefficient rank=0.115 and p=0.047), but not with subluxation.

Thermal sensitivity (41.9%) and occlusion (30.6%) are the most common symptoms of concussion and subluxation. Sensitivity to occlusion (p<0.0001) and mobility (p=0.03) were more related to the cases of subluxation than concussion (Table 4).

**Table 4:** Frequency of clinical signs in cases of concussion and subluxation

Clinical signs	Concussion		Subluxation		Total		P value
	n	%	n	%	n	%	
Spontaneous pain	4	8.2	33	13.1	37	12.3	0.33
Mobility	5	10.2	77	30.6	82	27.2	0.003*
Sensitivity to occlusion	4	8.2	88	34.9	92	30.6	0.0001*
Thermal sensitivity	24	49	102	40.5	126	41.9	0.27
Sensitivity to percussion	15	30.6	72	28.6	87	28.9	0.77
Dental darkening	1	2.0	4	1.6	5	1.7	0.59

* denotes statistically significant result

P values are for comparisons between the two groups, and χ2 or Fisher's exact test for appropriate categorical variables

In 8 (16.3%) cases of concussion and 68 (27.1%) of subluxation, pulp necrosis was diagnosed, and 68.4% occurred within 3 months (Table 5). However, the Spearman correlation test shows that the more time passes, the greater the occurrence of pulp necrosis (p≤0.0001). The upper teeth are those at greatest risk of necrosis (correlation coefficient rank −0.0190, p=0.001).

**Table 5:** Time between the injury and the diagnosis of pulp necrosis

Time (months)	Concussion		Subluxation		Total		P value
	n	%	n	%	n	%	
Less than 1	4	50	15	22.1	19	25	
1 to 2	1	12.5	32	47.1	33	43.4	
3 to 6	2	25	11	16.2	13	17.1	
7 to 12	0	0	5	7.4	5	6.6	
Over 12	1	12.5	5	7.4	6	7.9	
Total	8	100	68	100	76	100	0.67

* denotes statistically significant result

P values are for comparisons between the two groups, and χ2 or Fisher's exact test for appropriate categorical variables

The presence of pulp necrosis was positively correlated with motorcycle accidents (p=0.014), the occurrence of direct impact (p≤0.0001), crown fracture with pulp exposure (p≤0.0001), darkening of the crown (p=0.004), and spontaneous pain (p≤0.0001); and negatively correlated with the occurrence of indirect impact (p≤0.0001), completion of antibiotic therapy, dental splint, and occlusal adjustment.

Table 6 shows the main treatments applied in this sample. Endodontic treatment was necessary for 16.3% and 26.6% of cases of concussion and subluxation, respectively.

**Table 6:** Descriptive distribution of procedures performed in cases of concussion and subluxation

Procedures	Concussion		Subluxation		Total	
	n	%	n	%	n	%
Antibiotic therapy	1	12.5	18	26.5	19	25
Occlusal adjustment	0	0	7	10.3	7	9.2
Splint	2	25	20	29.4	22	28.9
Endodontic	8	10.7	67	89.3	75	98.7
Restoration	4	11.8	30	44.1	34	44.7

## Discussion

This retrospective research evaluated the evolution of concussion and subluxation cases; pulp necrosis appears more often from 0 to 6 months after the trauma. Direct impact, darkening of the crown, presence of spontaneous pain, and complicated crown fracture show a greater association with pulp necrosis in these cases.

Traumatic dental injuries, in some findings, are more common in men aged between 11 and 20 years^4^ ^,^ ^10^ ^,^ ^11^ ^,^ ^16^ ^,^ ^18^ ^,^ ^20^, whose etiology factor include the practice of more aggressive sporting activities because of their more violent behavior^17^ ^,^ ^21^. This study showed that among the cases of concussion and subluxation, the most affected age group was 10-20 years for both genders, the most predominant etiologies among men were bicycle accidents (41.6%) and sporting events (13.5%); while for women were bicycle accidents (41.4%) and fall from height (13.8%).

Among all participants, the cycling accident was the most common etiology followed by fall from height, car accident, sports and motorcycle accidents, probably because of the great number of bicycles in the region^18^ and participants’ age. For other studies, the most prevalent etiologies were domestic accidents, sports and fights, falls, and automobile accidents^10^ ^,^ ^11^ ^,^ ^16^ probably because of the different characteristics of the population.

Earlier reports described that maxillary incisors^10^ ^−^ ^12^ ^,^ ^16^ ^,^ ^21^ are the most traumatized teeth because of their prominence, which sometimes present themselves in a protrusive position^11^, confirming the data found.

Little attention is sometimes given to the kind of force that caused the trauma. Direct impacts cause a significant number of crown fractures as well as the roots associated with concussion and subluxation. This result shows a greater association of dental fractures with concussion than subluxation, even though there is a similar prevalence of direct impacts in both groups. These fractures absorb most of the forces that affect the tooth, reducing the damage to the periodontium insertion, somehow preventing tooth avulsion. However, young patients are more susceptible to traumas of dislocation than fractures due to the greater resilience of bone tissue, being generally the most affected population^3^ ^,^ ^15^.

Motorcycle accidents showed a direct correlation with concussion and not with subluxation, what can be explained, in most cases, by the indirect impacts resulting from the use of safety equipment, such as helmets, known to produce high impact trauma. This shows the importance of educational campaigns for greater traffic safety.

The clinical signs most observed in both injuries were sensitivity to thermal stimuli, occlusion and percussion, as well as mobility and spontaneous pain. According to Andreasen and Pedersen^2^ (1985), pain is usually reported during occlusion and mastication.

Injury to periodontium and pulp by concussion and subluxation is usually small, transient, and without serious consequences^2^ ^,^ ^5^ ^,^ ^6^ ^,^ ^8^ ^,^ ^9^ ^,^ ^16^. However, when there is concomitant crown fracture, mainly in teeth with complete root formation, the possibility of necrotic pulp increases, considering that, in general, the patients are young people with large dentinal tubules, which, when exposed, may lead to contamination of the pulp, especially when pulp exposure occurs^3^ ^,^ ^14^.

Regarding the diagnosis of pulp necrosis, there are different chronological standards for various types of dislocation. In cases of concussion and subluxation, pulp necrosis can be diagnosed in the first six months after the trauma^3^ ^,^ ^15^. In this study, we interpreted that the occurrence of indirect impact decreases the risk of pulp necrosis, as well as antibiotic therapy and splint and occlusal adjustment, although there was no statistically significant correlation.

Dental darkening has as etiologic factor the obliteration of the root canal and pulp necrosis^6^ ^,^ ^13^ ^,^ ^16^. In this study, the number of teeth with dental darkness was small (6.6%) when compared to the number of endodontically treated teeth (98.7%), emphasizing that this clinical sign must not be used as a unique parameter for the indication of endodontic treatment. More effective tests, such as for sensitivity and/or cavity, assist in the early diagnosis of pulp pathologies. Radiographic examination does not replace the tests abovementioned for the diagnosis and monitoring of trauma, being very suitable for the diagnosis of pulp necrosis and external root resorption, which is rare in this type of injury^9^.

We observed that dental splint and occlusal adjustment decrease the chance of experiencing tooth necrosis. Dental splint is dispensable for the cases of concussion and may be employed in subluxation for a short period of time (2 weeks); it is better to maintain the repositioned tooth in correct position, provide patient comfort and improved function^7^. In this study, we found that it was used for the concussion and subluxation due to more severe injuries occurring in the neighboring teeth.^12^ Thus, antibiotic therapy was also used because of other traumas in the neighboring teeth.

Occlusal adjustment is an important step, because premature contacts may occur even in small shifts, causing unwanted additional trauma^3^ ^,^ ^18^.

The association of subluxation and the presence of previous dental trauma may be explained by individual behavior and habits (i.e. kind of transport, sport practice), not by a deficiency in the repair of a previous injury.

This retrospective study highlighted the importance of knowledge of the dental trauma repair process in the diagnosis, treatment, monitoring, and prognosis of cases.

Possible limitations of this study include the reliance of the method on medical records, completed by different dental students and dental surgeons along these years, although the instructions were the same.

## Conclusion

Although concussion and subluxation traumas are considered of minor degrees, they must be followed to decrease the possibility of pulp necrosis and its early diagnosis and treatment in a timely manner favors a good prognosis.
